# Clinical, epidemiological, and laboratory characteristics of mild-to-moderate COVID-19 patients in Saudi Arabia: an observational cohort study

**DOI:** 10.1186/s40001-020-00462-x

**Published:** 2020-11-25

**Authors:** Abbas Al Mutair, Saad Alhumaid, Waad N. Alhuqbani, Abdul Rehman Z. Zaidi, Safug Alkoraisi, Maha F. Al-Subaie, Alanoud M. AlHindi, Ahmed K. Abogosh, Aljwhara K. Alrasheed, Aya A. Alsharafi, Mohammed N. Alhuqbani, Njoud A. Alhowar, Samer Salih, Mogbil A. Alhedaithy, Jaffar A. Al-Tawfiq, Haifa Al-Shammari, Rayid Abdulqawi, Alaa F. Ismail, Noura Hamdan, Fares Saad, Fahad A. Olhaye, Tarig A. Eltahir, Ali A. Rabaan, Awad Al-Omari

**Affiliations:** 1 Research Center, Dr. Sulaiman Al Habib Medical Group, Riyadh, Saudi Arabia; 2 University of Wollongong, Wollongong, Australia; 3 College of Pharmacy, King Saud University, Riyadh, Saudi Arabia; 4 Administration of Pharmaceutical Care, Alahsa, Ministry of Health, Rashdiah Street, P. O. Box 12944, Alahsa, 31982 Saudi Arabia; 5 College of Medicine, Alfaisal University, Riyadh, Saudi Arabia; 6 Department of Critical Care, Al Hammadi Hospital, Riyadh, Saudi Arabia; 7 Infectious Disease Unit, Specialty Internal Medicine, Johns Hopkins Aramco Healthcare, Dhahran, Saudi Arabia; 8 Department of Medicine, Indiana University School of Medicine, Indianapolis, IN USA; 9 Department of Medicine, Johns Hopkins University School of Medicine, Baltimore, MD USA; 10 Department of Histopathology, King Saud Medical City, Riyadh, Saudi Arabia; 11 Department of Internal Medicine, Al Hammadi Hospital, Riyadh, Saudi Arabia; 12 Molecular Diagnostics Laboratory, Johns Hopkins Aramco Healthcare, Dhahran, Saudi Arabia

**Keywords:** COVID-19, SARS-CoV-2, Symptoms, Comorbidities, Saudi Arabia, Epidemiology

## Abstract

**Background:**

Severe acute respiratory syndrome coronavirus 2 (SARS‐CoV‐2) emerged from China in December 2019 and has presented as a substantial and serious threat to global health. We aimed to describe the clinical, epidemiological, and laboratory findings of patients in Saudi Arabia infected with SARS-CoV-2 to direct us in helping prevent and treat coronavirus disease 2019 (COVID-19) across Saudi Arabia and around the world.

**Materials and methods:**

Clinical, epidemiological, laboratory, and radiological characteristics, treatment, and outcomes of pediatric and adult patients in five hospitals in Riyadh, Saudi Arabia, were surveyed in this study.

**Results:**

401 patients (mean age 38.16 ± 13.43 years) were identified to be SARS-CoV-2 positive and 80% of cases were male. 160 patients had moderate severity and 241 were mild in severity. The most common signs and symptoms at presentation were cough, fever, fatigue, and shortness of breath. Neutrophil and lymphocyte counts, aspartate aminotransferase, C-reactive protein, and ferritin were higher in the COVID-19 moderate severity patient group. Mild severity patients spent a shorter duration hospitalized and had slightly higher percentages of abnormal CT scans and X-ray imaging.

**Conclusions:**

This study provides an understanding of the features of non-ICU COVID-19 patients in Saudi Arabia. Further national collaborative studies are needed to streamline screening and treatment procedures for COVID-19.

## Background

In late 2019, a few unidentified pneumonia patients were found to have a formerly unfamiliar infection of a subcoronavirus, that was labeled 2019-novel coronavirus (2019-nCoV) [1]. This was later labeled by the World Health Organization (WHO) as severe acute respiratory syndrome coronavirus 2 (SARS-CoV-2) and the disease was branded coronavirus disease 2019 (COVID-19) [2]. Many countries have started their safety measures and started screening people coming into their country. Saudi Arabia started its precautions and implemented screening for travelers, along with an international travel ban until the disease is more controlled. A governmental move followed by several decisions was made to prevent the outbreak. Along with other guidelines, the Ministry of Health and the Saudi Center for Disease Prevention and Control published the Coronavirus Infection Guidelines [3] that were designed to promptly detect and treat COVID-19. As of August 24, 2020, the total reported confirmed COVID-19 cases have reached more than 308,600 including more than 3600 deaths within Saudi Arabia, and the increasing number of cases geographically with fatalities has raised multiple concerns [4]. Notwithstanding the rising amount of confirmed cases, the clinical data of SARS-CoV-2 patients in Saudi Arabia are insufficient.

Human to human transmission of the virus is now clear and well reported in many countries [5]. The mode of transmission for SARS-CoV-2 is mostly seen through respiratory droplets, direct contact with an infected individual, or by touching a contaminated surface or object [6, 7]. Precautions have to be taken including effective frequent handwashing and individuals should be keeping ≥ 6 feet away from other people are crucial in helping reduce the risk of infection and to halt the transmission cycle [8]. Currently, researchers from many countries are conducting clinical trials to produce or evaluate potential treatments, including a local ongoing trial to test the efficacy of convalescent plasma in our population [6]. However, we still do not have any approved treatments or fully ready vaccines for COVID-19 [7]. COVID-19 patients initially present with mostly fever, dry cough, and fatigue. A variety of less abundant symptoms may occur such as anosmia, hypogeusia, diarrhea, and headache [8]. In more severe cases, patients may have chest pain, dyspnea, and even loss of speech or movement [8]. The most challenging issue with COVID-19 is that a large fraction of infected individuals are asymptomatic carriers, which can also transmit SARS-CoV-2 to other individuals [9]. We aimed to describe the clinical, epidemiological, and laboratory features of patients in Saudi Arabia infected with SARS-CoV-2 to direct us in helping prevent and treat coronavirus disease 2019 (COVID-19) across Saudi Arabia and around the world.

## Materials and methods

### Study population

Between April 22nd and May 22nd, 2020, we enrolled 401 patients with COVID-19 at four branches of our private tertiary care hospital and one private hospital in Riyadh, Saudi Arabia.

### Design

A large team was assembled to collect data retrospectively from electronic medical records, and each record was then reviewed independently by four trained doctors.

### Data collection

Data gathered included demographic data, past medical history, symptoms, laboratory values, chest X-rays and CT scans, and the treatment provided. Laboratory values included complete blood counts, biochemistries, biomarkers for organ function, and analysis of immunological responses. Patients' severities were classified based on the “Chinese Clinical Guidance for COVID-19 Pneumonia Diagnosis and Treatment” published by the Chinese National Health Commission [10]. Mild clinical symptoms or asymptomatic with no signs of pneumonia in imaging were defined as “Mild”; and "Moderate" were characterized as having signs of pneumonia on imaging, and fever or clinical respiratory tract manifestations. The therapeutic principles comprised of general supportive therapy; active control over high fever; oxygen uptake if necessary; antiviral treatments; and monitoring of liver, kidney, myocardial, and lung functions. Several other investigational therapies evaluated for COVID-19 treatment (hydroxychloroquine, azithromycin, oseltamivir, vitamin C, vitamin E, ceftriaxone, and enoxaparin), were also included. The time taken to become SARS-CoV-2 PCR negative, duration of hospitalization, and treatment outcomes were recorded.

### Statistical analysis

Continuous variables were presented as mean (± SD) and categorical variables were written as number (%). We compared proportions for categorical variables between groups using Chi-square test. When values were normally distributed, we used independent group* t*-tests to compare means of continuous variables between groups; else, we used the Mann–Whitney *U* test. To test whether the observed heart rate, PR interval, QRS interval, and QTc interval during hydroxychloroquine treatment were statistically distinct from the baseline, paired samples* t*-test was used. We evaluated a *p*-value less than 0.05 as statistically significant. All data analyses were completed with SPSS version 25.

### Ethical considerations

This study was approved by the Institutional Review Board of Dr. Sulaiman Al Habib Medical Group and followed the Declaration of Helsinki. As patient data were deidentified and this was a retrospective study, written consent was not required.

## Results

Among the 401 hospitalized patients, 241 (60.1%) cases were separated into the mild group and 160 (39.9%) cases were split into the moderate group. The mean age of patients was 38.16 ± 13.43 years (median: 36.00) and 321 (80%) cases were male. Of these cases, only 4% of those patients were under 18 years of age. The main ethnicities represented in our cohort were Asian (49.9%) and Middle Eastern (48.1%), and, in lower proportions, African (1.2%) and European (0.7%). Most patients were treated at Habib Medical Group (HMG) Suwaidi Hospital (26.2%), HMG Rayan Hospital (25.4%), and Al Hammadi Hospital (24.2%) (Table 1).

**Table 1:** Clinical and epidemiological features of COVID-19 patients

Epidemiological data	Total (*n* = 401)	Mild cases (*n* = 241)	Moderate cases (*n* = 160)	*p*-value*
Female patients	80 (20%)	50 (20.7)	30 (18.8)	0.624
Male patients	321 (80%)	191 (79.3)	130 (81.3)	
Age, years (SD, range)	38.16 (13.43, 1–88)	37.32 (13.6, 1–83)	39.43 (13.1, 1–88)	0.112
Age ≤ 18 years	16 (4%)	12 (75)	4 (25)	
Race				
Middle eastern	193 (48.1)	104 (43.2)	89 (55.6)	0.06
Asian	200 (49.9)	133 (55.2)	67 (41.9)	
African	5 (1.2)	2 (0.8)	3 (1.9)	
European	3 (0.7)	2 (0.8)	1 (0.6)	
Admission location				
HMG Suwaidi Hospital	105 (26.2)	83 (34.4)	22 (13.8)	≤ 0.001
HMG Rayan Hospital	102 (25.4)	71 (29.5)	31 (19.4)	
HMG Takhassusi Hospital	44 (11)	33 (13.7)	11 (6.9)	
HMG Olaya Hospital	53 (13.2)	30 (12.4)	23 (14.4)	
Al Hammadi Hospital	97 (24.2)	24 (10)	73 (45.6)	
Frequency of PCR performed (SD, range)	2.56 (1.51, 1–10)	2.57 (1.48)	2.55 (1.56)	0.710
Symptoms				
Cough	215 (53.6)	102 (42.3)	113 (70.6)	≤ 0.001
Fatigue	104 (25.9)	50 (20.7)	54 (33.8)	0.004
Headache	65 (16.2)	38 (15.8)	27 (16.9)	0.768
Diarrhea	30 (7.5)	17 (7.1)	13 (8.1)	0.690
Shortness of breath	90 (22.4)	32 (13.3)	58 (36.3)	≤ 0.001
Body temperature, °C				
Fever upon admission was ≥ 38 °C	146 (36.4)	0	146 (91.3)	≤ 0.001
Fever during hospitalization was ≥ 38 °C	62 (15.5)	0	62 (38.8)	≤ 0.001
Sore throat	88 (21.9)	32 (13.3)	58 (36.3)	0.052
Muscle pain	57 (14.2)	22 (9.1)	35 (21.9)	≤ 0.001
Joint pain	36 (9)	12 (5)	24 (15)	0.001
Nausea	26 (6.5)	16 (6.6)	10 (6.3)	0.877
Rhinorrhea	29 (7.2)	18 (7.5)	11 (6.9)	0.822
Dysgeusia	22 (5.5)	13 (5.4)	9 (5.6)	0.921
Vomiting	16 (4)	9 (3.7)	7 (4.4)	0.748
Anorexia	13 (3.2)	9 (3.7)	4 (2.5)	0.494
Chest pain	3 (0.7)	2 (0.8)	1 (0.6)	0.816
Abdominal pain	4 (1)	3 (1.2)	1 (0.6)	0.541
Sputum production	30 (7.5)	13 (5.4)	1 (0.6)	0.051
Anosmia	19 (4.7)	13 (5.4)	6 (3.8)	0.448
Hyposmia	3 (0.7)	2 (0.8)	1 (0.6)	0.816
Sweating	2 (0.5)	2 (0.8)	0	0.248
Comorbidities				
Hypertension	59 (14.7)	26 (10.8)	33 (20.6)	0.006
Hyperglycaemia	40 (10)	20 (8.3)	20 (12.5)	0.169
Chronic kidney disease	1 (0.2)	1 (0.4)	0	0.415
Chronic heart disease	11 (2.7)	4 (1.7)	7 (4.4)	0.103
Chronic lung disease	15 (3.7)	9 (3.7)	6 (3.8)	0.994
Obesity				
Class I	34 (8.5)	16 (6.6)	18 (11.3)	0.220
Class II	10 (2.5)	5 (2.1)	5 (3.1)	–
Class III	2 (0.5)	2 (0.8)	0	–
Not obese	355 (88.5)	218 (90.5)	137 (85.6)	–
Smoking				
Non-smoker	366 (91.3)	216 (89.6)	150 (93.8)	0.349
Smoker	27 (6.7)	19 (7.9)	8 (5)	–
No. of cigarettes per day				
10–15 cigarettes per day	11 (40.7)	9 (47.3)	2 (25)	–
20 cigarettes per day	13 (48.1)	8 (42.1)	5 (62.5)	–
30–40 cigarettes per day	3 (11.1)	2 (10.5)	1 (12.5)	–
Former smoker	8 (2)	6 (2.5)	2 (1.3)	–
Dyslipidemia	19 (4.7)	8 (3.3)	11 (6.9)	0.101
Pregnant	1 (0.2)	0	1 (0.6)	0.182
Immunocompromised status	1 (0.2)	1 (0.4)	0	0.415
Laboratory tests				
White cell count, × 10^9^/L	6.56 (3.7)	6.84 (2.3)	6.14 (5.2)	0.069
Platelet count, × 10^9^/L	236.7 (75.3)	250.6 (79.3)	215.9 (63.7)	≤ 0.001
Neutrophil count, × 10^9^/L	4.2 (5)	4.0 (4.0)	4.4 (6.2)	0.425
Lymphocyte count, × 10^9^/L	2.9 (6.1)	2.63 (2.7)	3.3 (9.1)	0.346
Alanine aminotransferase, U/L	37.29 (28.8)	35.8 (26.2)	39.4 (32.1)	0.399
Aspartate aminotransferase, U/L	31.1 (20.5)	28.4 (18.6)	34.9 (22.4)	≤ 0.001
C-reactive protein, mg/L	16.99 (32.4)	11.1 (28.4)	28.6 (36.5)	≤ 0.001
D-dimer, mg/L	0.6 (1.6)	0.57 (0.98)	0.69 (2.1)	0.028
Lactate concentration, mmol/L	1.5 (0.5)	1.5 (0.57)	1.45 (0.5)	0.590
Ferritin (ng/ml)	290.5 (409.6)	201.6 (275.0)	445.2 (541.2)	≤ 0.001
Radiography				
Chest CT				
Abnormal	67 (16.7)	35 (52.2)	32 (47.7)	0.239
Normal	3 (0.7)	0	3 (100)	
Chest X-ray				
Abnormal	140 (34.9)	73 (52.1)	67 (47.8)	0.101
Normal	193 (48.1)	118 (61.1)	75 (38.8)	
Treatment				
Oxygen inhalation	32 (8)	6 (2.5)	26 (16.3)	≤ 0.001
Amount of oxygen provided (L)	4.03 (2.83)	4.8 (4.4)	3.8 (2.3)	0.746
Hydroxychloroquine	66 (16.5)	23 (9.5)	43 (26.9)	≤ 0.001
Azithromycin	193 (48.1)	74 (30.7)	119 (74.4)	≤ 0.001
Oseltamivir	134 (33.4)	47 (19.5)	87 (54.4)	≤ 0.001
Vitamin C	18 (4.5)	9 (3.7)	9 (5.6)	0.371
Vitamin E	17 (4.2)	8 (3.3)	9 (5.6)	0.262
Ceftriaxone	41 (10.2)	17 (7.1)	24 (15)	0.16
Enoxaparin	5 (1.2)	1 (0.4)	4 (2.5)	0.085
Prognosis				
Hospitalization	315 (78.6)	185 (76.8)	130 (81.3)	0.074
Transferred	18 (4.5)	8 (3.3)	10 (6.3)	
Discharged	68 (17)	48 (19.9)	20 (12.5)	
Days taken to be SARS-CoV-2 PCR-negative, (SD, range)	3.08 (1.9, 1–11)	3.11 (1.9, 1–11)	3.0 (2.0, 1–10)	0.845
Days of hospitalization, (SD, range)	8.3 (6, 1–42)	7.37 (5.5, 1–34)	9.71 (6.5, 1–42)	≤ 0.001

COVID-19 = coronavirus disease 2019. SARS-CoV-2 = severe acute respiratory syndrome coronavirus 2

Data are *n* (%) or mean (SD), unless otherwise indicated

^*^p values suggest the disparity between pediatric and adult patients with moderate clinical type with pneumonia and mild clinical type (asymptomatic or upper respiratory infection)

Compared to mild patients, moderate patients were older in age (mean age 37.32 ± 13.6 years (median: 36.00) vs 39.43 ± 13.1 (median: 39.00); *p* = 0.112) and were more likely to have underlying comorbidities, such as hypertension (33 [20.6%] vs 26 [10.8%]), diabetes (20 [12.5%] vs 20 [8.3%]), chronic heart disease (7 [4.4%] vs 4 [1.7%]), chronic lung disease (6 [3.8%] vs 9 [3.7%]), obesity class I (18 [11.3%] vs 16 [6.6%]), obesity class II (5 [3.1%] vs 5 [2.1%]), dyslipidemia (11 [6.9%] vs 8 [3.3%]), and pregnancy (1 [0.6%] vs 0). Mild COVID-19 severity patients smoked more tobacco than moderate severity cases (7.9% vs 5%), mostly smoking 10–15 cigarettes per day (47.3%) or 20 cigarettes per day (42.1%) (Table 1).

The most common signs and symptoms at the onset of illness were cough (215 [53.6%]), fever ≥ 38 °C upon admission (146 [36.4%]), fatigue (104 [25.9%]), shortness of breath (90 [22.4%]), sore throat (88 [21.9%]), headache (65 [16.2%]), fever ≥ 38 °C during hospitalization (62 [15.5%]) and muscle pain (57 [14.2%]). Less common symptoms were joint pain (36 [9%]), diarrhea (70 [7.5%]), sputum production (30 [7.5%]), rhinorrhea (29 [7.2%]), nausea (26 [6.5%]), dysgeusia (22 [5.5%]), and anosmia (19 [4.7%]) (Table 1).

Abnormal laboratory findings in mild and moderate severity patients were C-reactive protein (CRP) (11.1 vs 28.6 mg/L), D-dimer (0.57 vs 0.69 mg/L) and ferritin (201.6 vs 445.2 ng/ml). Neutrophil count (4.4 vs 4.0 × 109/L), lymphocyte count (3.3 vs 2.6 × 109/L), alanine aminotransferase (39.4 vs 35.8 U/L), aspartate aminotransferase (34.9 vs 28.4 U/L), CRP (28.6 vs 11.1 mg/L), D-dimer (0.69 vs 0.57 mg/L), and ferritin (445.2 vs 290.5 ng/ml) were higher in the COVID-19 moderate severity patient group. Some laboratory tests differed significantly between the two severity groups, including increased aspartate aminotransferase (*p* ≤ 0.001), raised CRP (*p* ≤ 0.001), high levels of D-dimer (*p* = 0.028) and ferritin (*p* ≤ 0.001) (Table 1).

Generally, mild severity patients had slightly higher percentages of abnormal CT scans (52.2% vs 47.7%) and abnormal X-ray imaging (52.1% vs 47.8%) compared to moderate severity cases (Table 1). Over the course of treatment, moderate severity patients required supplementary oxygen via a nasal cannula in comparison to the mild severity patient group (16.3% vs 2.5%) and were administered higher amounts of hydroxychloroquine (26.9% vs 9.5%), azithromycin (74.4% vs 30.7%), oseltamivir (54.4% vs 19.5%), vitamin C (5.6% vs 3.7%), vitamin E (5.6% vs 3.3%), ceftriaxone (15% vs 7.1%), and enoxaparin (2.5% vs 0.4%) (Table 1).

Compared with the moderate severity group, the mean days taken to become SARS-CoV-2 PCR-negative was a bit higher in the mild severity patient group (mean 3.11 days vs 3.0 days, *p* = 0.845; respectively). Mild severity patients spent a shorter duration of hospitalization (mean 7.37 days vs 9.71 days, *p* ≤ 0.001; respectively). Mild severity patient group had better prognosis outcomes in terms of hospitalization (76.8% vs 81.3%), transfer (3.3% vs 6.3%), and discharge (19.9% vs 12.5%) (Table 1).

An aggregate of 63 patients had both an ECG recording at baseline and days 3–5 post initiation of hydroxychloroquine therapy. Table 2 shows the mean QTc interval prior to and throughout hydroxychloroquine treatment in all mild and moderate COVID-19 patients. Hydroxychloroquine treatment ensued in a mean QTc prolongation of 5.93 ms in all patients (95% CI 13.35–1.49 ms; *p* = 0.113) using computerized interpretation. Those receiving hydroxychloroquine in the mild COVID-19 severity group had a greater mean difference in QTc interval prolongation (8.5 [− 20.22 to 3.22] ms; *p* = 0.143) compared with those receiving hydroxychloroquine in the moderate severity group (2.5 [− 11.87 to 6.87] ms; *p* = 0.569). Not any of these patients had a prolonged interval ahead of the initiation of hydroxychloroquine therapy and none were using antiarrhythmic drugs simultaneously.

**Table 2:** Effect of hydroxychloroquine on QTc interval

	Patient COVID-19 severity type	Mean QTc before hydroxychloroquine treatment (ms) (95% CI)	Mean QTc during hydroxychloroquine treatment (ms) (95% CI)	Mean difference (ms) (95% CI)	*p*-value
Computer interpreted	All	400.68 (384.99–416.36)	414.06 (389.4–423.81)	5.93 (− 13.35 to 1.49)	0.113
	Mild	414.06 (392.61–435.52)	422.56 (397.85–447.28)	8.5 (− 20.22 to 3.22)	0.143
	Moderate	382.83 (360.85–404.81)	385.33 (364.89–405.77)	2.5 (− 11.87 to 6.87)	0.569

*CI *confidence interval

## Discussion

This is a large cohort study of hospitalized patients with SARS-CoV-2 in Saudi Arabia. There was no significant disparity in the ratio of female and male patients, and infection in children was exceptionally low, which was consistent with the findings of other studies [1, 8, 9].

The median age of our cohort was 36 years, which is identical to the discoveries of a national study, indicating that COVID-19 affects a younger age group in Saudi Arabia compared to the rest of the world [11]. This might be attributed mainly to the differences in the inclusion criteria and the population age groups in our study. However, all the age groups might have been infected, including those younger than 2 and older than 80. Our findings concur with previous studies, showing that patients infected with SARS-CoV-2 may present primarily with cough, fever ≥ 38 °C, lethargy, sore throat, shortness of breath, headache, and muscle pain with accompanying symptoms of sputum production, rhinorrhea, diarrhea, dysgeusia and anosmia like common cold [8, 9].

Mild severity patients may present without a fever and without signs of pneumonia; moderate patients usually have a fever ≥ 38 °C or respiratory symptoms, mainly shortness of breath, cough, and sore throat. There are many similarities between COVID-19, SARS-CoV, and new microorganisms such as avian influenza virus H7N9. However, there are also key differences. COVID-19 cases can vary from mild to moderate or even severe, while SARS cases or avian influenza virus H7N9, in general, were more severe [12]. Unlike SARS-CoV infections which usually result in a high-grade fever at the early onset of infection [12], some COVID-19 patients included in our report presented with atypical symptoms and only low-grade fever and a long incubation period leading to a higher potential for COVID-19 viral transmission and greater infectiousness.

In our study, abnormal chest X-ray findings were more than twice as common as chest CT abnormalities in all patients (34.9% vs 16.7%). Since some of the mild severity patients were asymptomatic, it has been proposed that a CT examination should be the earliest choice in the screening and diagnosis of COVID-19. This is because the sensitivity of CT scan for SARS-CoV-2 was found to be 98%, compared to the RT-PCR sensitivity of 71% [13]. Swift detection of COVID-19 is vital for disease therapy and control, and hence, a chest CT may be a more dependable, useful, and quick method to diagnose and evaluate COVID-19 [14].

As Al-Omari, et al. had recently pointed out, we deemed it necessary to investigate the laboratory and radiological features of COVID-19 patients in the Saudi population [15]. Our mild and moderate COVID-19 patients had elevated inflammatory markers (e.g., CRP, D-dimer, and ferritin), similar to cytokine release syndrome, with persistent fevers [16, 17]. A recent study from Saudi Arabia also had similar findings with higher CRP, D-dimer, ferritin, and glucose levels in moderate severity patients [3]. At the initial stage of COVID-19, the level of inflammation and lung lesions were positively linked with CRP levels, and thus, CRP levels could signify disease severity and were suggested to be utilized as a major marker for disease monitoring [18]. Recently, Wang, et al. also demonstrated that a CRP finding of > 26.9 mg/L could be used as a predictive marker for aggravating severity of COVID-19 [19]. An elevated level of D-dimer in patients with COVID-19 disease may provoke an emergent thrombotic complication and may due to the hyperactivation of the coagulation cascade. There is a systemic inflammatory response triggered by viral infections that can cause an imbalance between anticoagulant and procoagulant homeostatic processes [20]. Ferritin is a fundamental mediator of immune dysregulation and contributes to the cytokine storm via direct immune-suppressive and pro-inflammatory effects [21].

Higher neutrophil absolute count has been related to a greater severity in COVID-19 as well as lymphopenia [21, 22]; higher levels of lymphocytes has not. Moreover, higher levels of liver enzymes were more manifested in the moderate patients compared to the mild cases, indicating that liver inflammation and liver damage in moderate patients are more evident [1]. However, additional studies in Saudi Arabia are required to precisely identify which laboratory markers can potentially predict outcomes of COVID-19 patients in the Saudi population.

The ideal approach to the treatment of SARS-CoV-2 is uncertain and is based on limited data and evolves rapidly as clinical data emerge. Lack of a defined optimal management plan for COVID-19 disease results in the use of various treatment options and adjuvant therapies during hospital stay. For patients with non-severe disease, care is primarily supportive, with close monitoring for disease progression. Supportive measures offered to all patients in this study included the prevention of secondary infections, respiratory support, circulatory support, and preservation of renal, hepatic, and neurological function. In addition to the implementation of the basic principles of critical care medicine, patients received pharmacologic prophylaxis for venous thromboembolism. The frequencies of the supportive measures used for mild and moderate severity patient groups were not similar, but no conclusions can be made about efficacy. Hydroxychloroquine was used partly to treat the patients in both groups even though its routine use is not suggested outside the circumstances of a clinical trial given the lack of clear benefit from limited data and potential for cardiotoxicity [23, 24]. Given the lack of clear benefit and potential for toxicity, use of hydroxychloroquine in hospitalized patients is not suggested [25]. However, hydroxychloroquine was used when the data about its benefit were scarce, considering that we have accumulated enough information now to ensure that hydroxychloroquine is not effective as a COVID-19 treatment or prophylaxis.

Our analysis demonstrates that therapy of COVID-19 patients with hydroxychloroquine resulted in a non-statistically significant effect on QTc interval. Hydroxychloroquine inhibits voltage-gated sodium and potassium channels, prolonging the QT interval, and is also structurally analogous to the class IA antiarrhythmic quinidine [8]. Changes in QTc and prolongation findings due to hydroxychloroquine use aligned with previous studies of substantial QTc prolongation in 11–23% of patients [26, 27]. While the addition of azithromycin to hydroxychloroquine might have been implicated in QTc prolongation [28], it is still conceivable that the genuine degree of QTc prolongation associated with hydroxychloroquine was estimated imprecisely, given patient severity and characteristics variation and a limited follow-up period.

It is pertinent to recognize some limitations of this study. First, the retrospective study design could have introduced potential reporting bias due to reliance on clinical case records. Second, without a control group, we were unable to determine that hydroxychloroquine and azithromycin increases cardiotoxic risk; but, compared with solely hydroxychloroquine, changes in QTc differences were likely related to the addition of azithromycin. Furthermore, we were not able to provide details on the radiological characters of our SARS-CoV-2-infected patients. Moreover, many of the study patients are still hospitalized at the time of the writing of this manuscript. Consequently, there may have been some partiality regarding the prognosis of the patients. Finally, some follow-up data were unavailable. Clinical follow-up data for patients after recovery from SARS-CoV-2 infection could be used to examine longer-term functional and psychological abnormalities.

## Conclusions

Although fever, cough, fatigue, and shortness of breath are common symptoms, more than half of patients have neither clear signs nor abnormal radiological findings. The large number of asymptomatic patients suggests difficulty in identifying some COVID-19 patients. In contrast to the mild cases, the moderate ones had higher neutrophil counts, lymphocyte counts, alanine aminotransferase, aspartate aminotransferase, CRP, and higher levels of ferritin, but lower levels of D-dimer, lactate concentrations, and platelet counts. Chest CT scans ought to be the earliest choice for the screening and diagnosis of COVID-19 due to higher sensitivity. There are currently no fully certified treatments for COVID-19 and with the help of ongoing clinical trials and further international collaborative studies, we will be able to develop first-class screening and treatment protocols.

## Data Availability

The data used to support the findings of this study are available from the corresponding author upon request.

## Abbreviations

COVID-19: Coronavirus disease 2019
SARS-CoV-2: Severe acute respiratory syndrome coronavirus 2
RT-PCR: Real-time reverse transcription-polymerase chain reaction
CRP: C-reactive protein
